# Enhancing Fritillariae Cirrhosae Bulbus authentication via Mask-AIR: a masked autoencoder driven information reconstruction and iterative attention framework for multi-view fusion

**DOI:** 10.3389/fnut.2026.1928119

**Published:** 2026-10-05

**Authors:** Huan Du, Xiu Gu, Hai Zhu, Yu Chen, Xin Wang, Xiaoqin Zhi, Ziqin Lei, Xiaohui Qu, Gang Fan

**Affiliations:** 1 Department of Pharmacy, Affiliated Hospital of Southwest Jiaotong University, The Third People's Hospital of Chengdu, Chengdu, Sichuan, China; 2 Department of Pharmacy, Sichuan Provincial People's Hospital East Sichuan Hospital & Dazhou First People's Hospital, Dazhou, China; 3 Department of Otolaryngology Head and Neck Surgery, West China Hospital, Sichuan University, Chengdu, China; 4 School of Life Science and Engineering, Southwest Jiaotong University, Chengdu, China; 5 School of Ethnic Medicine, Chengdu University of Traditional Chinese Medicine, Chengdu, China; 6 Center for Digital Intelligence and Innovation (CDII), Chengdu University of Traditional Chinese Medicine, Chengdu, China

**Keywords:** authenticity identification, Fritillariae Cirrhosae Bulbus, functional foods, hyperspectral classification, iterative attention, masked autoencoder, multi-view fusion

## Abstract

Fritillariae Cirrhosae Bulbus (FCB), a valuable medicinal and edible plant with antitussive properties, is frequently adulterated due to resource scarcity and high market price, necessitating rapid and accurate authentication. However, existing methods (morphological, molecular, chromatographic, etc.) are subjective, destructive, or suffer from DNA degradation. To address these limitations, this paper proposes a multi-modal information reconstruction framework based on a masked autoencoder (MAE) and an iterative attention mechanism that integrates a multi-view fusion strategy using three views (hyperspectral imaging, grayscale images, and pseudo-colour images) to enhance the authenticity identification of FCB. Specifically, multi-view data are fused to enrich information dimensions and thereby improve species recognition accuracy. An MAE operating in the latent feature space reconstructs randomly masked tokens from each modality, which enhances feature representation and mitigates cross-modal interference. Subsequently, hierarchical iterative attention module alternates query roles among modalities to balance fusion. Feature extraction, reconstruction, and classification are trained end-to-end, while inference retains only the encoder and classifier, incurring no extra cost. The experiments on a self-built FCB multi-view dataset show that the proposed method significantly improves classification accuracy across all categories and outperforms both conventional and deep learning baselines. Therefore, this work provides an effective non-destructive tool for FCB authenticity identification and can be extended to the authentication of other valuable functional foods.

## 1 Introduction

Fritillariae Cirrhosae Bulbus (FCB, Chuanbeimu) is a botanical ingredient widely used in both traditional medicine and functional foods, known for its antitussive and expectorant properties. Its therapeutic efficacy has led to widespread clinical use, and its commercial importance is evidenced by products such as the Fritillaria-based cough syrup Nin Jiom Pei Pa Kao (Fritillaria Loquat Syrup), which generates annual retail sales exceeding 1.8 billion RMB in China and is distributed in over 20 countries (1, 2). Beyond pharmaceuticals, Fritillaria species are also used in functional foods (e.g., Fritillaria and pear soup) and ornamental horticulture (3). However, the growing market demand has placed immense pressure on wild resources. The highest-quality FCB are primarily wild-harvested from high-altitude, cold zones, and over-exploitation has led to a sharp decline in natural populations (4). Consequently, the wild FCB and *Fritillaria pallidiflora* (FPB) have been listed as endangered plant species in China. Compounding this scarcity, the market price of FCB is approximately 10 to 200 times higher than that of other *Fritillaria* species (2). Such a vast price differential, driven by profit motives, has made adulteration rampant: merchants frequently substitute or mix the valuable herb with cheaper look-alikes, including *Fritillaria ussuriensis* (Pingbeimu), *Fritillaria thunbergii* (Zhebeimu), *F. pallidiflora* (Yibeimu), and even non-medicinal bulbs. These practices not only deceive consumers and compromise therapeutic efficacy but also pose safety risks, as some adulterants are similar-appearing yet toxic species. Moreover, post-harvest processing practices such as sulfur fumigation and mechanical grinding, which are commonly employed to improve appearance and facilitate storage, further exacerbate these authentication challenges by altering the physical and chemical characteristics of the samples, thereby masking critical diagnostic features and increasing the difficulty of reliable identification. Therefore, effective and practical technological approaches are urgently needed to accurately authenticate FCB, particularly its most valuable commercial grade “Songbei,” and to distinguish it from its adulterants.

Preliminary investigations by our research group have found that substantial research has been devoted to the authentication of Fritillaria species, yielding a diverse array of methodological approaches (5). These range from macroscopic and microscopic morphological inspection to molecular techniques (e.g., DNA barcoding), spectroscopic technologies, chromatographic methods (including thin-layer chromatography and high-performance liquid chromatography), sensor-based assays, and mass spectrometry. Despite its simplicity, morphological inspection suffers from inherent subjectivity and loses its discriminatory power when applied to processed materials, such as sliced or powdered bulbs. Although molecular techniques offer high species specificity, they are frequently compromised by DNA degradation resulting from post-harvest processing or sulfur fumigation, which leads to unreliable or false-negative results (6). Spectroscopic and sensor-based methods, while non-destructive, require extensive calibration and are sensitive to sample heterogeneity. Chromatographic and mass spectrometric techniques, on the other hand, deliver accurate quantification but are destructive, labor-intensive, and dependent on expensive instrumentation as well as skilled operators. In summary, each of these existing methods suffers from at least one critical drawback, which could be subjectivity, destructiveness, time consumption, or vulnerability to pre-analytical factors such as sulfur fumigation, and these drawbacks make them unsuitable for rapid, on-site, and large-scale screening. This situation thus motivates the exploration of alternative non-destructive technologies that can overcome these limitations while delivering reliable and efficient authentication.

Non-destructive testing (NDT) technologies, such as near-infrared spectroscopy and hyperspectral imaging (HSI), have emerged as attractive alternatives due to their ability to preserve sample integrity, enable rapid analysis, and support high-throughput screening (7, 8). Among these, HSI has emerged as a particularly powerful tool, as it integrates two-dimensional imaging with spectroscopy to simultaneously acquire both spatial and spectral information at the pixel level (9). This unique combination enables HSI to capture detailed biochemical and physical profiles of samples, making it well-suited for detecting adulteration, identifying species and geographical origin, and evaluating compositional attributes (10). Moreover, HSI requires minimal sample preparation and avoids the use of chemical reagents during analysis, substantially reducing chemical waste and environmental impact (11). However, the practical deployment of these NDT technologies, including HSI, is constrained by several inherent limitations. Signal reproducibility is often compromised by sample heterogeneity, moisture content, and surface irregularities, which introduce variability in spectral acquisition (12). Moreover, the interpretation of high-dimensional spectral data requires sophisticated chemometric or machine learning models, whose generalisability remains questionable when applied to new batches or different environmental conditions (13). The absence of standardised protocols for data acquisition, preprocessing, and model validation further hampers cross-study comparison and regulatory acceptance. For botanical ingredients such as FCB, these issues are compounded by morphological similarities between authentic and adulterated species, the prevalence of processed or powdered forms, and spectral degradation caused by sulfur fumigation. These limitations highlight the need for more advanced strategies that can leverage the strengths of NDT while overcoming its current constraints.

In recent years, deep learning coupled with hyperspectral imaging has achieved considerable success across diverse fields, including agriculture, environmental science, and medical imaging. For instance, a multi-path residual attention network (MRA-Net) with chunked residual channel attention was developed for duct cancer diagnosis using hyperspectral images under limited training data, achieving 84.31% accuracy on a public dataset, demonstrating the potential of HSI for pathological analysis even with small samples (14). Another major emerging research direction is the application of hyperspectral imaging technology combined with machine learning in the quality control of herbal medicine from the perspective of artificial intelligence (15). Nevertheless, the deployment of HSI-based deep learning in real-world scenarios still faces two major challenges: the high spatial variability of spectral features and the scarcity of large, standardised labelled datasets for training. Recognising these difficulties, an increasing number of studies have turned to the integration of additional views or modalities to capture more comprehensive feature information, thereby boosting classification performance. A U-Net-based feature extraction backbone has been proposed to learn multi-scale invariant features from multi-view data, which are subsequently combined with a Transformer network to facilitate information capture and modeling (16). Another study introduced the MTFFN network, which employs two branches to transfer dual-view knowledge from RGB images and source HSI data to the target domain, using a multi-task learning strategy for feature fusion (17). Furthermore, unsupervised encoder-decoder structures have been explored, where co-training of two unsupervised features in a latent subspace, guided by limited labelled samples, yields discriminative multi-view representations (18). These developments suggest that embracing multi-modal and multi-view information, particularly through strategies that reconstruct and reinforce feature representations in a latent space, may offer a viable path forward. Such an approach may offer a way to reduce the reliance on large labelled datasets, mitigate cross-modal interference, and enhance fusion-based identification robustness, thereby helping to alleviate some of the current difficulties in authenticating FCB and its adulterants.

Despite these advances, existing multi-view fusion methods typically adopt simple feature concatenation or shared-decoder reconstruction strategies, which often overlook two critical issues. First, cross-view interference occurs when a dominant view suppresses complementary information from weaker views during fusion. Second, conventional attention-based fusion usually assigns a fixed view as the query, which exacerbates this imbalance and fails to dynamically adapt to varying sample conditions. Moreover, the inherent challenges in acquiring and annotating hyperspectral data mean that these models must operate under limited labelled samples, yet few existing methods fully exploit the intrinsic structural information within each view through self-supervised pre-training. In this work, building on the existing masked autoencoder (MAE) and attention mechanisms, we address these limitations through three task-specific innovations and their coordinated integration. First, we propose view-specific masked reconstruction in the latent feature space using independent decoders with unshared weights, which allows each view to learn robust representations without cross-view contamination, a strategy that has not previously been explored in HSI-based MAE frameworks. Second, we introduce an iterative cross-attention strategy that alternately assigns different views as queries during successive fusion stages, dynamically balancing their contributions and effectively mitigating modal dominance. Third, these modules are jointly optimised end-to-end, while the reconstruction decoders are removed during inference, imposing no additional computational burden. Specifically, Mask-AIR performs view-specific masked reconstruction in the latent feature space using independent decoders with unshared weights, thereby enhancing feature representations while reducing cross-view interference. Furthermore, an iterative cross-attention strategy is introduced to alternately assign different views as queries during successive fusion stages, preventing a single view from dominating the fusion process and enabling more balanced integration of complementary information. These modules are jointly optimized during training, while the reconstruction decoders are removed during inference, thus imposing no additional computational burden. In addition, we focused on “Songbei,” the most precious commercial grade of FCB, and built a deep learning dataset consisting of this authentic grade along with its common adulterant, sulfur-fumigated “Xiaopingbei.” Finally, extensive experiments conducted on the collected FCB multi-view hyperspectral dataset demonstrate that our method significantly improves classification accuracy across all categories, thereby validating the effectiveness of the proposed model. Consequently, this work provides a powerful tool for the authenticity identification of FCB and offers a reference approach for the authentication of other valuable herbs.

## 2 Materials and methods

### 2.1 Samples preparation

In this study, two batches of *Fritillaria* samples were collected. These consisted of 180 Songbei (Fritillariae Cirrhosae Bulbus) grains, which were sourced from the Songpan region of Sichuan, and 250 Xiaopingbei (Fritillariae Ussuriensis Bulbus) grains, which originated from Liaoning province in northeastern China. Although both species belong to the same genus, Xiaopingbei is frequently used as a counterfeit for the more valuable Songbei, because its sulfur-fumigated products closely resemble Songbei in appearance after processing, and the market price of Songbei is substantially higher than that of Xiaopingbei, which makes adulteration economically attractive. The most notable morphological distinction between the two lies in the central bulb, which in Xiaopingbei not only exhibits a slightly blackish coloration but also differs in its morphological characteristics compared to that of Songbei. All samples were authenticated by Professor Hongxiang Yin at Chengdu University of Traditional Chinese Medicine and were found to comply with the standards of the 2025 Chinese Pharmacopoeia. Based on Professor Yin’s identification records, we manually annotated the dataset to establish the ground-truth labels for authentic FCB versus adulterant Xiaopingbei. The samples were stored in the laboratory of the College of Ethnic Medicine, Chengdu University of Traditional Chinese Medicine. Hyperspectral data were acquired in reflectance mode using a portable hyperspectral imaging system (iSpecHyper-VS1000, Lisen Optics, China) equipped with a Basler acA1920-50gm detector. The system covered 400–1,000 nm with 300 spectral bands and a spectral resolution of approximately 2.1 nm. Two 1,000 W halogen lamps were symmetrically positioned at 45° relative to the sample-surface normal. During acquisition, the integration time was 62,501 μs, the frame rate was 16 fps, the scanning-angle range was 50°–73°, and the spatial resolution was 480 × 624 pixels. After processing, 430 samples were obtained, each consisting of three views: the complete hyperspectral cube (HSI), a grayscale image (GREY) independently captured using a conventional camera, and a pseudo-RGB image derived from the hyperspectral cube by retaining and stacking the first three principal components. To evaluate the performance of the proposed model, we adopted k-fold cross-validation, in which the dataset was equally partitioned into five subsets. In each fold, one subset was designated as the test set, while the remaining four subsets were used for training. After completing five rounds of model evaluation, the results were obtained by averaging the outcomes across all folds.

### 2.2 Implementation details

All training and testing procedures were performed within a Python 3.8 environment on the Ubuntu 20.04.4 LTS operating system, utilizing the PyTorch 1.5 framework for model construction and training. The training and evaluation processes were conducted on a workstation equipped with an Nvidia RTX 3090 graphics card, which provides 24 GB of video memory, a memory clock of 19.5 GHz, a 384-bit memory bus, and a memory bandwidth of 936 GB/s. Throughout the training phase, the proposed algorithm was trained on the hyperspectral multi-view dataset of *Fritillaria cirrhosa* for 100 epochs, with a batch size of 64 per iteration. The optimization was carried out using the Adam optimizer in conjunction with a step-wise learning rate decay schedule. Specifically, the learning rate was reduced from its initial value of 5e−3 to 5e−4 at the 20th epoch, with a decay factor (gamma) of 0.9. The weight decay of Adam was set to 5 × 10^−3^, and the remaining parameters used the default settings, including $\beta_{1}=0.9$ , $\beta_{2}=0.999$ , and $\epsilon =10^{-8}$ . Owing to the limited number of samples, we employed 5-fold cross-validation with random sampling, in which all samples were randomly divided into five mutually exclusive subsets, and for each fold, four subsets were used for training while the remaining subset served for testing. To evaluate the performance of the proposed network and to enable comparison with other methods, three widely adopted performance metrics were employed, namely overall accuracy (OA), average accuracy (AA), and the Kappa coefficient, the detailed formulations of which are provided in Equations 1–3. Among them, True Positive (TP)

$$\mathrm{OA}=\frac{\sum_{i=1}^{C}\;TP_{i}}{\sum_{i=1}^{C}\;N_{i}}$$ (1)

$$\mathrm{AA}=\frac{1}{C}\sum_{i=1}^{C}\;\frac{TP_{i}}{N_{i}}$$ (2)

$$p_{0}=\frac{\sum_{i=1}^{r}\;X_{\mathrm{ii}}}{N};p_{e}=\frac{\sum_{i=1}^{r}\;X_{\mathrm{ir}}\times X_{\mathrm{ic}}}{N^{2}};\text{Kappa}=\frac{p_{o}-p_{e}}{1-p_{e}}$$ (3)

refers to the number of samples that are actually positive and correctly predicted as positive; True Negative (TN) refers to the number of samples that are actually negative and correctly predicted as negative; False Positive (FP) denotes the number of samples that are actually negative but predicted as positive; and False Negative (FN) denotes the number of samples that are actually positive but predicted as negative. In the matrix, *X_ii_* represents the diagonal entries (correctly classified samples for class *i*), *X_ir_* denotes the total observed counts in row *i*, and *X_ic_* denotes the total observed counts in column *i*. Here, p *_e_* represents the proportion of observed accuracy, indicating the correctly classified samples; while p _0_ represents the proportion of chance agreement, indicating misclassifications due to random factors.

### 2.3 Basic architecture of Mask-AIR

Mask-AIR employs a masked autoencoder (MAE) to enhance the feature representation capability of different views through information reconstruction in the latent space. Enhancing view-specific features also helps mitigate the influence of other views on a given view during multi-view information fusion. In designing the feature enhancement model, we further analyze how varying the number of tokens in the encoder and decoder of the U-Net architecture affects the quality of information reconstruction and feature enhancement. To promote multi-view fusion, we improve upon existing attention mechanisms by introducing a hierarchical iterative attention fusion method. This method effectively addresses the inefficiency of cross-view fusion caused by excessively high intra-view attention scores, thereby boosting model performance when multiple data sources are used. The feature extraction, information reconstruction, and classification modules are trained jointly in an end-to-end fashion. We adopt a simple structure to achieve feature enhancement while suppressing interference from multi-view fusion and enhancing classification capability. Notably, during inference, the model retains only the feature extraction and classification modules, ensuring that the information reconstruction module imposes no additional computational burden on inference speed.

#### 2.3.1 Feature extractor module

The convolutional neural networks (CNNs) have demonstrated remarkable potential in modeling spatial relationships among features for hyperspectral image (HSI) recognition tasks. Let the HSI data is denoted as $H_{s}\in {ℛ}^{W\times H\times B}$ , where *W* and *H* represent the spatial dimensions (width and height, respectively) and *B* denotes the number of spectral bands. Other modalities are uniformly represented as *H_o_*. The large number of spectral bands *B* provides rich information, enabling CNNs to effectively model complex spatial relationships and allowing the U-Net architecture to investigate the influence of varying token counts on feature quality. This capability to capture both spectral and spatial features is essential for projecting the broad spectral bands of HSI into a high-level space while preserving sufficient information for subsequent analysis in the MAE reconstruction module.

$$H_{s},H_{o}=\text{Conv}2D(\text{Conv}3D((H_{s},H_{o})))$$ (4)

$${\hat{H}}_{s},{\hat{H}}_{o}=\text{DSconv}(H_{s},H_{o})$$ (5)

In the proposed model (Figure 1), a 3D convolutional layer is first applied to extract spatial and spectral features from the hyperspectral data. This operation serves to reduce the dimensionality of the spectral bands while retaining critical spectral-spatial information, thereby making the data more tractable for further processing. Following the 3D convolution, a 2D convolutional layer is employed to extract high-level features that remain stable across different data views. The combination of 3D and 2D convolutions constitutes the core of our feature extraction pipeline, enabling efficient capture of spectral-spatial information while ensuring that the extracted features are robust and adaptable to various input views. Finally, as part of the U-Net architecture, a downsampling convolution reduces the dimensions of *Hs* and *H_o_* to a fixed number of tokens *N*, facilitating subsequent processing and analysis as expressed in Equations 4, 5.

**Figure 1:**
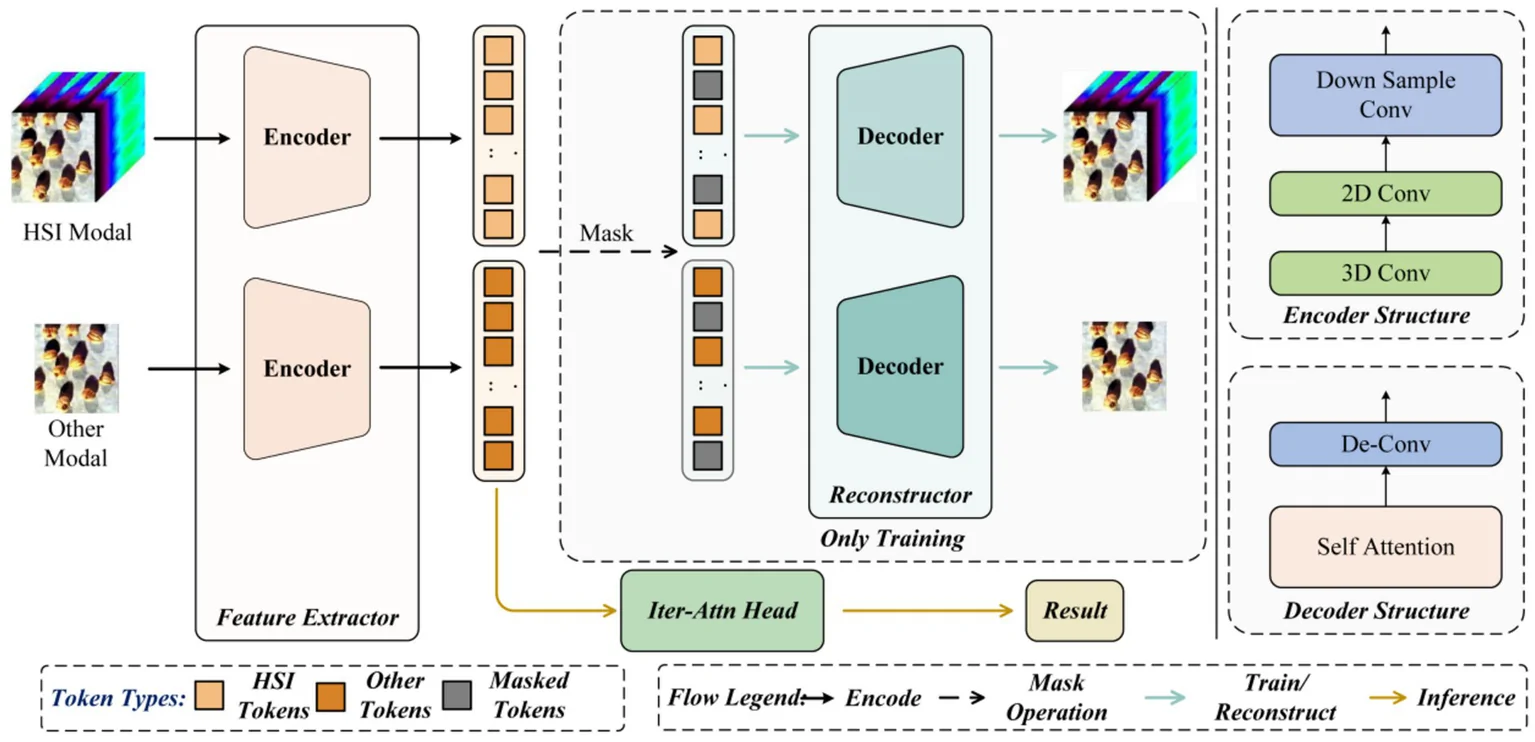
Architecture of the masked autoencoder driven information reconstruction and iterative attention framework (MAE-AIR).

#### 2.3.2 MAE reconstruction module

To achieve optimal fusion of multi-view data while preserving the unique characteristics of each view and reducing information interference among views, this chapter designs a masked MAE reconstruction module, whose structure is illustrated in Figure 2. Unlike the standard MAE, this module performs reconstruction only in the multi-view feature space rather than in the original input space, thereby retaining the extracted spatial and spectral features while avoiding the negative impact of excessive fusion on individual view features.

**Figure 2:**
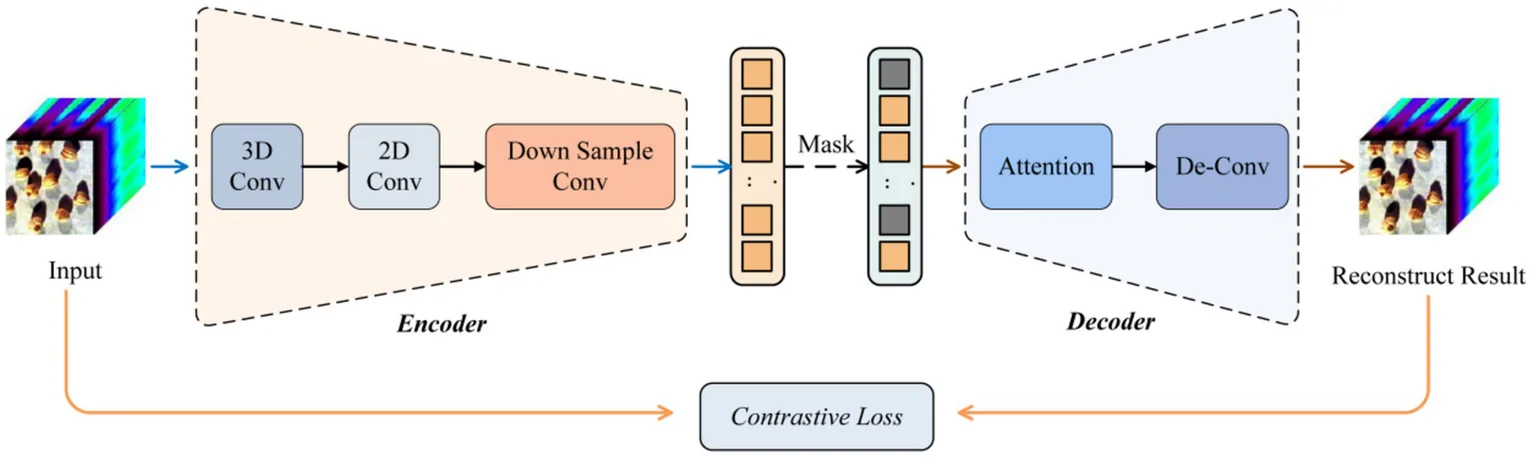
MAE-based multi-view feature reconstruction module.

Specifically, the feature extractor serves as the encoder, while masked decoders operate on the output feature representations of the hyperspectral view and other views, denoted as ${\hat{H}}_{s}\;$ and $\;{\hat{H}}_{o}$ respectively. Each decoder generates random mask tokens for its corresponding view to reconstruct its intrinsic information. The decoders share the same architecture across views but do not share weights. Each decoder consists of a series of Transformer blocks, with the final layer being a linear projection that restores the reconstructed feature space to the same spatial and spectral dimensions as the input data. This design enables the model to independently reconstruct and enhance the feature representation of each view, effectively preserving the uniqueness and integrity of each view while alleviating information interference in multi-view fusion. In the experiments, following the MAE setup, feature tokens are randomly masked at a ratio of 75%. The decoder predicts the feature values of the masked tokens. The reconstruction loss is mean squared error (MSE), applied only to the masked positions. This module is enabled only during training and disabled during testing, thus incurring no additional inference overhead and significantly improving model efficiency.

#### 2.3.3 Iterative attention head for multi-modal fusion

As shown in Figure 3, a cross-attention (CA) mechanism is iteratively applied to gradually fuse multi-view information. In each view branch, this mechanism distinguishes between relevant information and interference, thereby enhancing the representation of useful features. Specifically, the standard attention mechanism from Transformers is adopted, which evaluates similarity among different views by computing attention scores. This enables the model to dynamically assess the relative importance of each view, mitigating imbalances caused by excessively high attention scores in a single view and ensuring a balanced and efficient fusion process.

**Figure 3:**
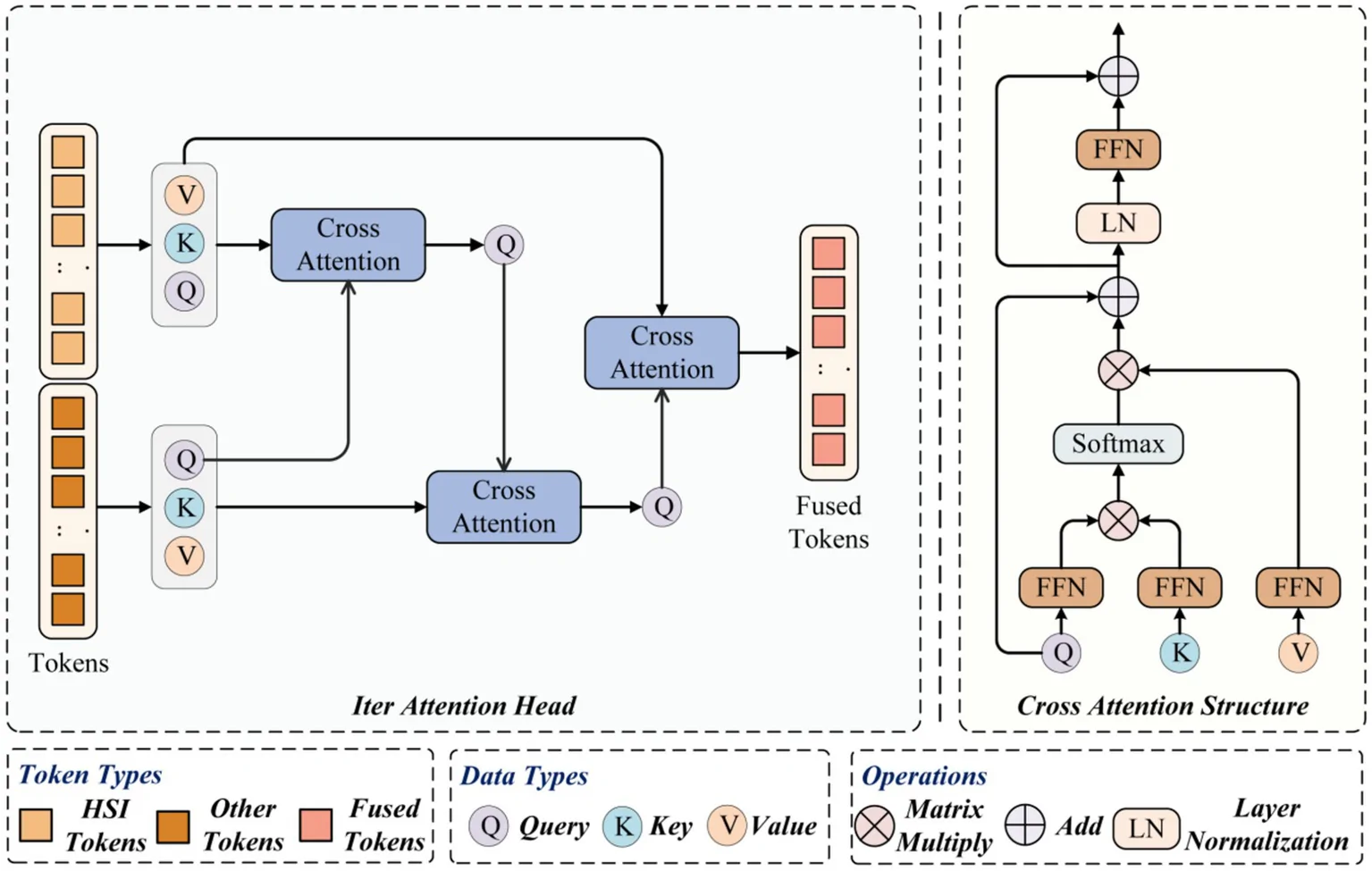
Iterative cross-attention mechanism for progressive multi-view fusion and balanced feature integration.

Unlike traditional methods such as ViT (19), which directly concatenate feature tokens from all views before feeding them into a Transformer, the proposed Iter-Attn method explicitly accounts for the possibility that one view may dominate others through an iterative, view-specific attention mechanism. By iteratively evaluating the importance of each view, this approach effectively alleviates imbalance among views. The specific form of the cross-attention mechanism is given in Equations 6, 7.

$${\mathrm{CA}}_{1}=\text{Softmax}(\frac{\mathrm{FFN}(Q)\mathrm{FFN}(K)}{\sqrt{d_{k}}})\mathrm{FFN}(V)+Q$$ (6)

$${\mathrm{CA}}_{2}=\mathrm{FFN}(\mathrm{LN}({\mathrm{CA}}_{1}))+{\mathrm{CA}}_{1}$$ (7)

Among them, *Q*, *K*, and *V* denote the query, key, and value, respectively, while *d_k_* represents the dimension of the keys. FFN refers to a single layer of a feed-forward network, and LN denotes layer normalization of the features. In our method, the cross-attention mechanism is divided into two stages, namely CA_1_ and CA_2_, where the query information is iteratively used to enhance its representation. Specifically, as shown in the left part of Figure 3, ${\hat{H}}_{o}\;$ is first used as the query, with ${\hat{H}}_{s}$ serving as both the key and value. The output of this stage is then reconsidered as the query for the ${\hat{H}}_{o}$ branch, where $\;{\hat{H}}_{o}$ now serves as the key and value. In the final stage, the updated output is again used as the query, but the key and value sources are switched to ${\hat{H}}_{s}$ .

Through this iterative process, the fused representation is repeatedly selected as the query, enabling the attention map to capture relationships among different views. The resulting attention scores reflect the combined response of the fusion, rather than the influence of any single view. The ultimate goal is to enhance the classification capability across all views. To evaluate this, the output of the Iter-Attn Head is used to assess classification performance, and the cross-entropy loss is applied, as expressed in Equation 8.

$$L_{\mathrm{cls}}=-\sum_{i}(y_{i}^{\mathrm{gt}}\log(y_{i})+(1-y_{i}^{\mathrm{gt}})+\log(1-y_{i}))$$ (8)

where $y_{i}^{\mathrm{gt}}$ is the ground-truth label of the i-th sample, and $y_{i}$ is the predicted probability for the positive class.

## 3 Results

In this section, a series of experiments are conducted to validate the proposed multi-view fusion recognition algorithm based on information reconstruction, including ablation studies and comparative experiments on the algorithm architecture. First, parameter sensitivity analysis is performed to investigate the effect of different feature token counts on the MAE information reconstruction module, and ablation experiments assessed the impact of both the MAE module and the iterative attention module on recognition accuracy. Finally, a comprehensive comparison is made between the proposed multi-view fusion information reconstruction framework and other state-of-the-art methods to verify the effectiveness of our model in the hyperspectral species identification of FCB.

### 3.1 Influence of multi-view information on the classification performance of FCB

To evaluate the influence of different view combinations on the authenticity identification performance of FCB, four input strategies were designed, as shown in Table 1. These include single-view input using only HSI, two-view inputs using HSI + GREY and HSI + PCA, and three-view input using HSI + PCA + GREY. The experimental results show that the best single-view result (OA 93.95%, AA 93.87%, Kappa 87.16%) is noticeably lower than any two-view combination, indicating that relying solely on spectral information has inherent limitations. Among the two-view inputs, the HSI + GREY combination approaches near-saturation performance with OA reaching 99.06%, suggesting that grayscale images provide strongly complementary information. Even though the performance is already close to the upper bound, the remaining marginal benefit from further model improvement is limited. In contrast, the highest OA of the HSI + PCA combination is 96.28%, which is markedly lower than that of HSI + GREY, indicating that pseudo-colour images contribute limited yet still exploitable discriminative information. The three-view fusion (HSI + PCA + GREY) achieves the best overall performance (OA 99.53%, AA 99.04%, Kappa 99.01%), but the improvement over the best two-view combination is less than 0.5 percentage points, implying that adding a third view yields only marginal gains. In summary, multi-view fusion can effectively enhance the authenticity identification performance of FCB, with grayscale images contributing significantly more than pseudo-colour images. Nevertheless, when complementary information is already abundant, further adding views may lead to diminishing returns. In practice, the choice of view combination should be balanced against data availability and cost-effectiveness.

**Table 1:** Results of ablation experiments on model architecture under different data modalities.

Modality	Attention	Reconstruction	OA (%)	AA (%)	Kappa (%)
HSI	CAT	NONE	88.98 ± 1.67	89.36 ± 2.74	79.26 ± 1.99
HSI	CAT	MAE	90.70 ± 2.06	89.29 ± 2.23	80.02 ± 2.47
HSI	ITER	NONE	90.88 ± 1.26	88.31 ± 2.08	82.28 ± 2.11
HSI	ITER	MAE	93.95 ± 1.93	93.87 ± 2.10	87.16 ± 2.83
HSI + GREY	CAT	NONE	96.74 ± 1.49	96.67 ± 2.08	93.14 ± 1.77
HSI + GREY	CAT	MAE	99.06 ± 0.41	99.01 ± 1.17	98.03 ± 1.71
HSI + GREY	ITER	NONE	99.06 ± 0.54	98.79 ± 1.69	98.02 ± 1.55
HSI + GREY	ITER	MAE	99.06 ± 0.46	98.79 ± 1.72	98.02 ± 1.13
HSI + PCA	CAT	NONE	92.70 ± 1.47	91.86 ± 1.55	88.64 ± 1.98
HSI + PCA	CAT	MAE	95.95 ± 0.94	94.61 ± 1.83	92.98 ± 1.56
HSI + PCA	ITER	NONE	94.49 ± 1.88	93.46 ± 1.92	93.02 ± 1.38
HSI + PCA	ITER	MAE	96.28 ± 1.05	95.85 ± 0.84	93.11 ± 1.99
HSI + PCA + GREY	CAT	NONE	97.30 ± 0.54	96.85 ± 1.84	96.12 ± 1.53
HSI + PCA + GREY	CAT	MAE	99.06 ± 0.33	98.79 ± 0.67	98.02 ± 0.37
HSI + PCA + GREY	ITER	NONE	99.06 ± 0.42	99.01 ± 0.36	98.04 ± 0.65
HSI + PCA + GREY	ITER	MAE	99.53 ± 0.25	99.04 ± 0.41	99.01 ± 0.59

### 3.2 The effect of features and model structure on classification performance

The effect of different feature token counts on algorithm performance was investigated through a parameter sensitivity analysis, the results of which are presented in Figure 4. The number of feature tokens directly affects the information reconstruction capability of the MAE-based module, thereby influencing the overall classification performance. In this figure, the x-axis represents the number of feature tokens, while the y-axis denotes the OA. Hyperspectral data differ from other views in that they contain substantially more dimensional information; consequently, conventional sampling parameters used in CNNs for ordinary images are not directly applicable to HSI data. To address this issue, an ablation study based on feature token count was designed to determine the optimal dimensionality of the reduced features extracted by the feature extraction network. Dimensionality reduction is achieved through a downsampling convolution layer within the CNN-based multi-view feature extraction module. The original data dimension is 11 × 11 (corresponding to 121 tokens). By adjusting the parameters of this convolution, four additional token counts (16, 36, 64, and 100) are obtained, corresponding to feature dimensions of 4 × 4, 6 × 6, 8 × 8, and 10 × 10, respectively. Including the original dimension, a total of five feature token counts are evaluated. As can be observed, the optimal performance for all input view combinations is achieved when the token count is set to 64. Furthermore, the results clearly demonstrate that fusing all three views yields the best recognition performance.

**Figure 4:**
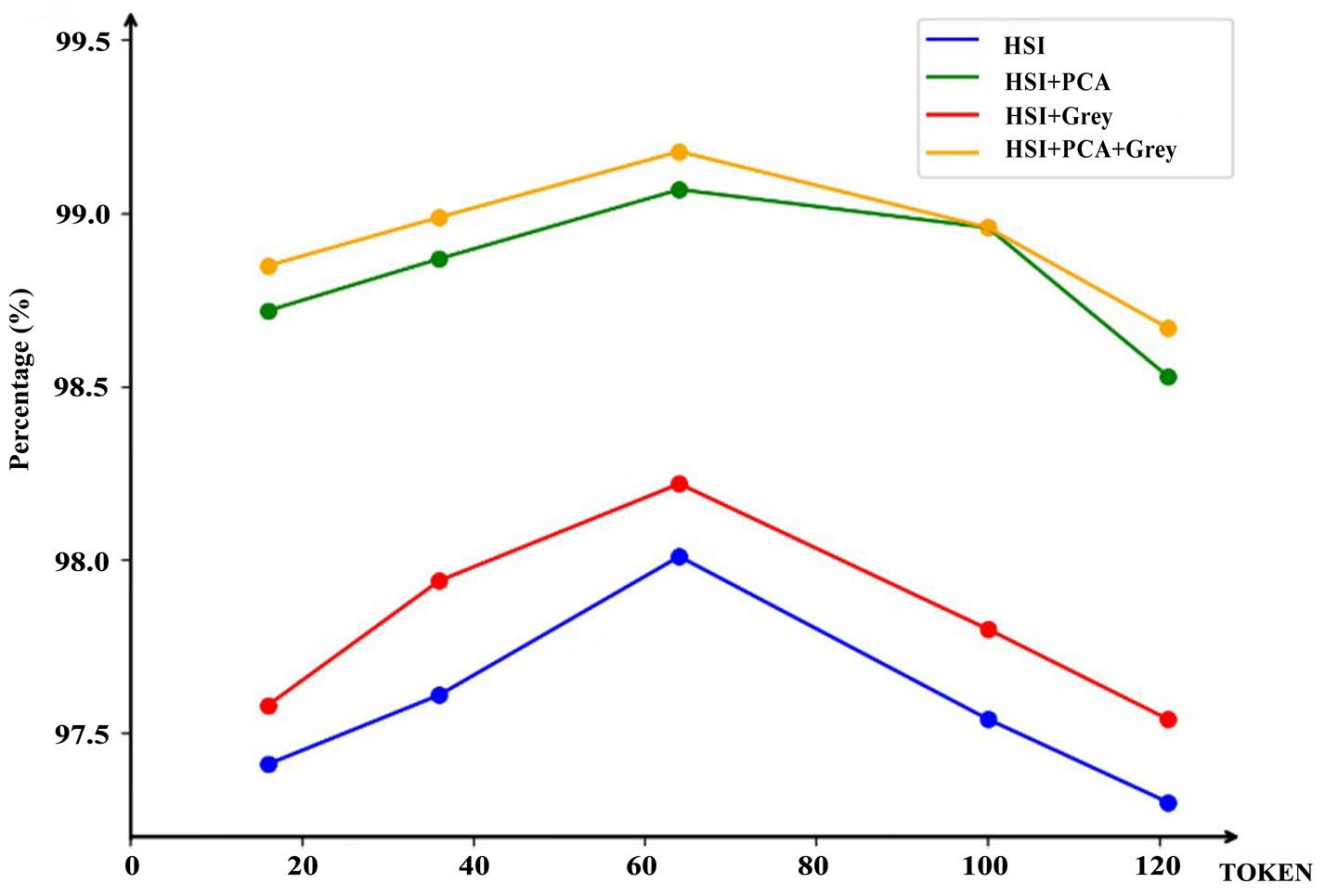
Effect of feature token quantity on model performance.

A set of ablation experiments were conducted to evaluate the effectiveness of the proposed information reconstruction module and the iterative attention mechanism under different input combinations, as summarized in Table 1, Figure 5. Four model configurations were compared, namely CAT (concatenation of multi-source features followed by a standard attention mechanism), ITER (the proposed multi-branch iterative attention module), MAE (information reconstruction applied during training), and NONE (training without reconstruction). The results consistently show that ITER outperforms CAT, as ITER facilitates progressive cross-source interactions and more effective stepwise fusion. Likewise, incorporating MAE improves classification performance compared to training without reconstruction.

**Figure 5:**
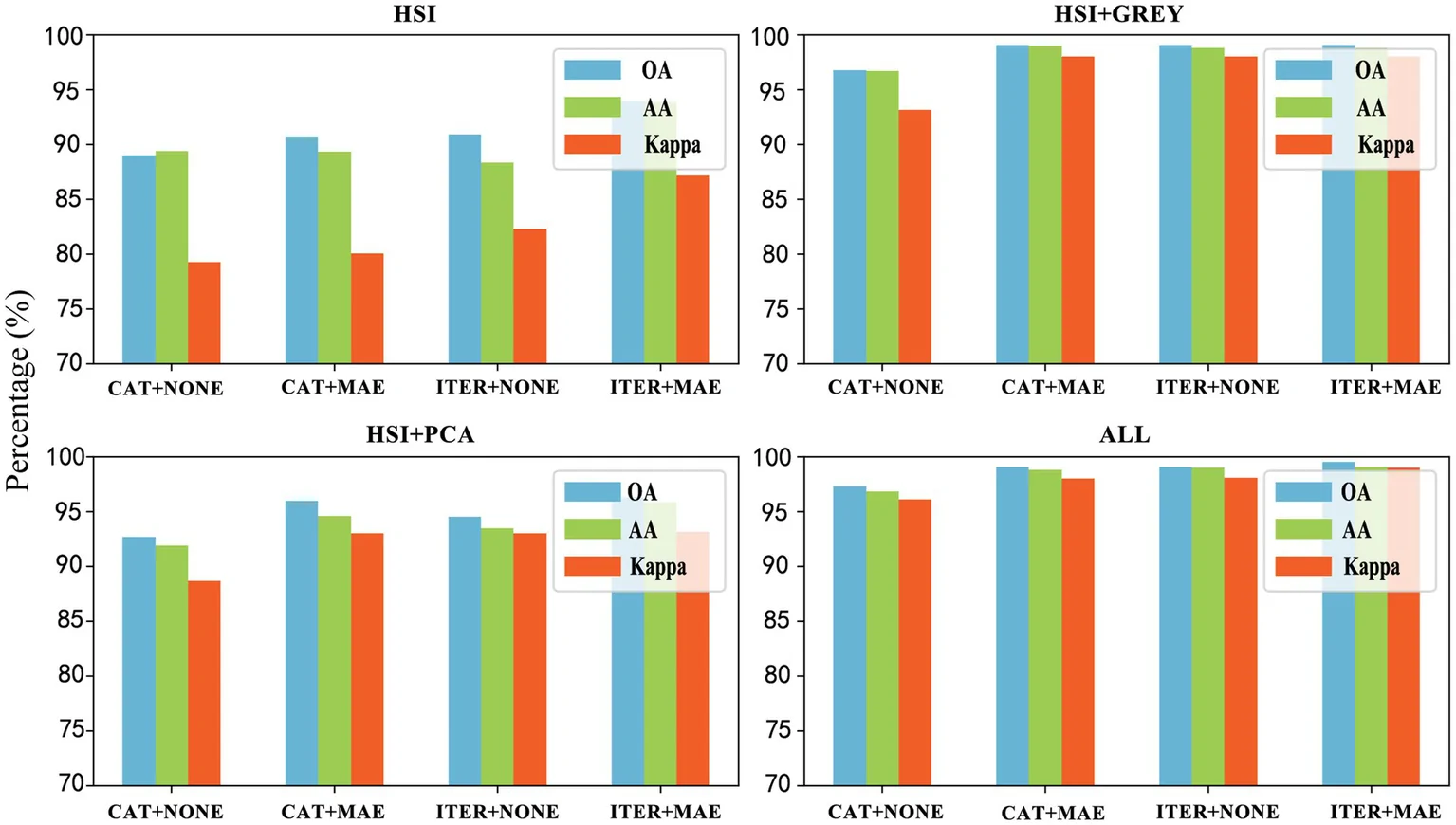
Ablation study of the proposed information reconstruction module and iterative attention mechanism under different data modalities.

For single-source input using only hyperspectral data (HSI), the combination of ITER and MAE achieves the best performance (OA 93.95%, AA 93.87%, Kappa 87.16%), while the baseline CAT with NONE yields only 88.98, 89.36, and 79.26%, respectively. The corresponding improvements of 4.97, 4.51, and 7.90 percentage points are the most substantial among all input combinations, indicating that when the original information is limited, iterative attention and feature-space reconstruction provide the greatest marginal benefit. For the two-source input HSI + GREY, which supplies strongly complementary information, performance differences among configurations become marginal: CAT + MAE, ITER + NONE, and ITER + MAE all reach OAs close to 99.06%, with ITER + MAE showing no further advantage. This suggests that when complementary information is already abundant, the gain from refining model structures tends to converge. For the HSI + PCA combination, where complementary information is weaker, ITER + MAE still exhibits a clear improvement over CAT + NONE (an OA increase of 3.58 percentage points), confirming the effectiveness of the proposed modules in weak-information scenarios. When all three sources are fused (HSI + PCA + GREY), ITER + MAE achieves the overall best performance (OA 99.53%, AA 99.04%, Kappa 99.01%), yet the improvement over the best HSI + GREY result (99.06% OA) is less than 0.5 percentage points. This indicates that under high information redundancy, adding a third source yields only a marginal gain. In summary, iterative attention and information reconstruction play a critical role when data are insufficient or complementary information is weak, whereas their marginal benefit diminishes as information becomes abundant. Therefore, the choice of model configuration and input sources should be balanced against the specific data conditions and performance requirements in practice.

### 3.3 Comparison of the identification performance of different deep learning classification models on the FCB dataset

The proposed multi-view fusion information reconstruction model is compared with conventional CNNs [CNN2D (20) and CNN3D (21)] and transformer-based methods [ViT (19), SpectralFormer (22), EfficientSAM (23) and EoMT (24)] using the same dataset. The OA, AA, and Kappa coefficient for identifying “Songbei” and “Xiaopingbei” are used as evaluation metrics with the results presented in Figure 6, Table 2. Among the conventional convolutional methods, CNN3D achieves the best performance with an OA of 93.95%, an AA of 92.62%, and a Kappa of 86.98%. However, its extensive use of 3D convolution leads to high computational overhead, which is unfavorable for real-time inference. Transformer-based methods generally outperform convolutional counterparts, as exemplified by ViT and SpectralFormer, which achieve OAs of 94.78 and 95.28%, respectively. Among recent Transformer-based approaches, EfficientSAM achieves an OA of 98.86%, an AA of 97.58%, and a Kappa of 96.32%, whereas EoMT attains 99.61, 99.18, and 98.58%, respectively. Our Mask-AIR_HGP surpasses EfficientSAM by 0.67% in OA, 1.46% in AA, and 2.69% in Kappa, while also yielding competitive results against EoMT. Notably, the proposed model using only the single-view HSI (Mask-AIR_H) attains an OA of 93.95% and an AA of 93.87% where the AA even exceeds that of ViT by 0.81%. This indicates that even without auxiliary views, information reconstruction and iterative attention can still provide competitive feature representations although the absolute performance remains lower than any model employing auxiliary views. This suggests inherent limitations of relying solely on spectral information. Multi-view fusion significantly improves performance. Mask-AIR_HG (HSI + GREY) reaches 99.06% OA. Mask-AIR_HGP (three views) achieves the best results with an OA of 99.53%, an AA of 99.04%, and a Kappa of 99.01%. These values are 4.75, 5.98, and 9.88% higher than those of ViT and 4.25, 4.74, and 7.82% higher than those of SpectralFormer, respectively. This validates the positive role of the information reconstruction module in feature learning and the key contribution of iterative attention in cross-view fusion. It should be noted that the OA of Mask-AIR_HP (HSI + PCA) is only 96.28% which is markedly lower than that of Mask-AIR_HG. This indicates that the discriminative information provided by PCA-based pseudo-RGB images is weaker than that of grayscale images, although it is still exploitable.

**Figure 6:**
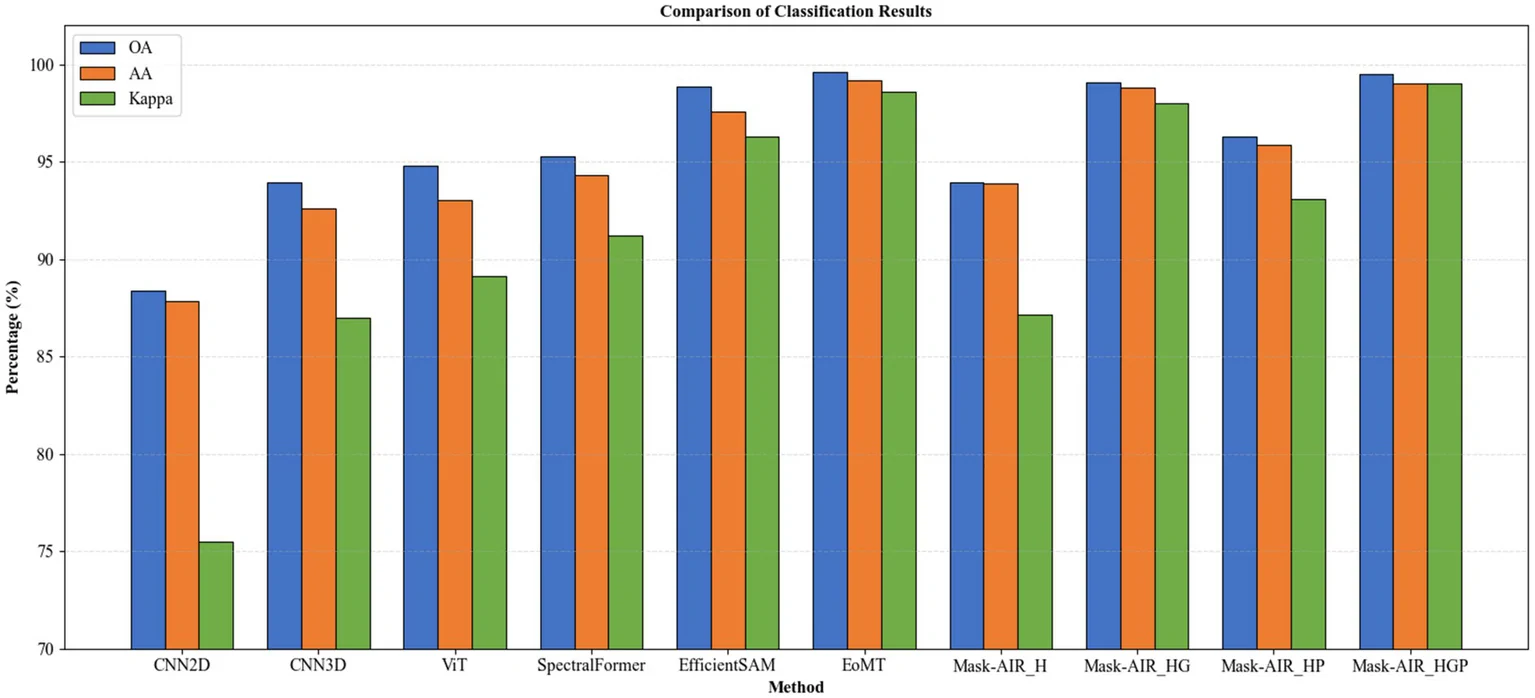
Performance comparison of different deep learning methods for authenticity identification of FCB.

**Table 2:** Performance comparison of different deep learning classification models for authenticity identification of FCB.

Method	Songbei (%)	Xiaopingbei (%)	OA (%)	AA (%)	Kappa (%)
CNN2D	85.54 ± 1.82	90.15 ± 2.88	88.37 ± 2.43	87.85 ± 2.13	75.52 ± 1.10
CNN3D	89.43 ± 2.94	97.48 ± 1.65	93.95 ± 1.59	92.62 ± 1.25	86.98 ± 2.18
ViT	92.77 ± 2.03	96.21 ± 1.40	94.78 ± 1.98	93.06 ± 1.44	89.13 ± 2.28
SF	92.57 ± 1.75	96.93 ± 0.80	95.28 ± 1.38	94.30 ± 1.52	91.19 ± 1.09
EfficientSAM	99.18 ± 0.39	98.05 ± 1.33	98.86 ± 0.94	97.58 ± 1.02	96.32 ± 1.03
EoMT	99.72 ± 0.15	99.34 ± 0.38	99.61 ± 0.31	99.18 ± 0.44	98.58 ± 0.45
Mask-AIR_H	91.20 ± 1.62	96.54 ± 1.84	93.95 ± 2.02	93.87 ± 1.40	87.16 ± 1.34
Mask-AIR_HG	99.56 ± 0.29	98.02 ± 1.23	99.06 ± 0.66	98.79 ± 1.33	98.02 ± 0.47
Mask-AIR_HP	96.05 ± 0.46	95.65 ± 1.67	96.28 ± 0.49	95.85 ± 0.57	93.11 ± 0.83
Mask-AIR_HGP	100.00 ± 0.00	98.08 ± 0.68	99.53 ± 0.22	99.04 ± 0.74	99.01 ± 0.62

To verify the effectiveness of the proposed algorithm in practical applications, a set of “Songbei” and “Xiaopingbei” samples with similar appearances was selected and authenticated by three experts in herbal medicine identification from Chengdu University of Traditional Chinese Medicine, with the test results for FCB authenticity identification shown in Figure 7. Comparing the detection outcomes of CNN3D, ViT, SpectralFormer, and the proposed model reveals that when the morphological similarity between samples is high (for example, samples No. 5, 7, and 12), most methods produce misclassifications. Nevertheless, Transformer-based methods generally outperform conventional CNN3D. The proposed model (Mask-AIR_HGP) fuses three views and employs an MAE in feature space for feature enhancement. It also improves the attention mechanism to better integrate cross-view information. Consequently, it achieves completely correct classification even for highly similar samples.

**Figure 7:**
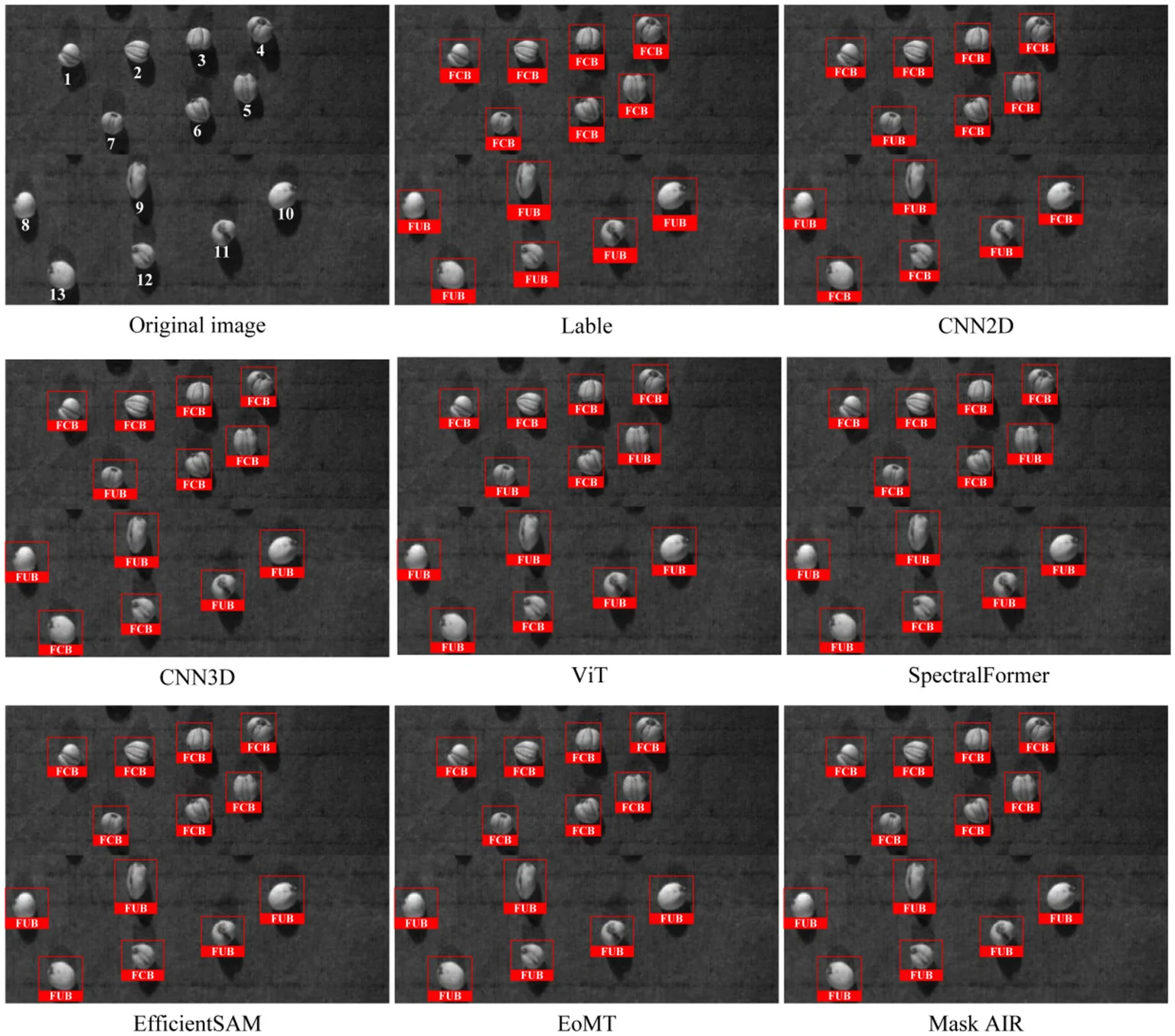
Qualitative performance comparison of different deep learning algorithms on the authentic and adulterated FCB dataset.

In summary, multi-view fusion can effectively enhance the authenticity identification performance of FCB, where grayscale images contribute significantly more than pseudo-colour images. Iterative attention and information reconstruction are particularly beneficial when data are scarce, whereas their marginal gains diminish when view information is already abundant. Even so, the proposed model demonstrates leading performance in both quantitative and qualitative evaluations, providing an effective tool for the rapid, non-destructive authentication of valuable herbs.

## 4 Discussion

### 4.1 Overall assessment of methodological effectiveness

The proposed Mask-AIR method achieves excellent classification performance on the self-built multi-view hyperspectral dataset of FCB. Experimental results demonstrate that by fusing three complementary views, namely hyperspectral images (HSI), grayscale images, and pseudo-RGB images generated via principal component analysis (PCA), the method attains an overall accuracy (OA) of 99.53%, an average accuracy (AA) of 99.04%, and a Kappa coefficient of 99.01%, significantly outperforming conventional CNN methods as well as existing Transformer-based models such as ViT (19) and SpectralFormer (22). These results validate the effectiveness of the proposed multi-modal information reconstruction framework for authenticating high-value botanical ingredients, while also offering a novel approach to addressing common challenges in hyperspectral classification, such as limited labelled data, cross-modal interference, and spectral variability. Moreover, the method retains only the feature extraction and classification modules during inference, imposing no additional computational burden, which makes it promising for practical deployment.

It is worth emphasising that the successful development of Mask-AIR relies on a thorough understanding of the design principles of the masked autoencoder (MAE) and their appropriate adaptation to feature-space reconstruction tasks. As a scalable visual self-supervised learning framework, the MAE learns representations by randomly masking image patches and reconstructing the missing pixels. The asymmetric encoder-decoder architecture proposed by He et al. (25), in which the encoder processes only the visible patches while the decoder receives both the latent representations and the mask tokens, is key to achieving efficient large-scale self-supervised pre-training. However, migrating this framework to a multi-modal hyperspectral scenario requires careful design at several levels.

To begin with, the asymmetric encoder-decoder design is embodied in our framework by the fact that the MAE operates only on the tokens output by the feature extractor rather than on the raw input. Moreover, a high masking ratio of 75% is retained so that the reconstruction task remains sufficiently challenging in feature space to drive semantic learning, which is consistent with the original MAE core design that raises the masking ratio to 75% to create a meaningful self-supervised task. Furthermore, the choice of reconstruction target is critical. Existing studies have pointed out that directly using raw pixels or features as reconstruction targets in remote sensing images may cause the model to over focus on pixel-level details and become sensitive to noise (26). In response, researchers have proposed various improvements, such as using image features instead of the original image as the reconstruction target (26), introducing inductive reconstruction losses to create a more difficult masked image modelling task (27), or employing dynamic background reconstruction strategies to address the difficulty of distinguishing between foreground and background differences (28). In addition, the synergistic design of the masking strategy and multi-view fusion is another key aspect that distinguishes our framework from traditional MAE. Using only a single MAE makes it difficult to effectively capture complementary relationships across modalities. To address this issue, existing work has explored a two-stream MAE framework to reconstruct masked spatial patches and spectral channels separately, adopted a multi-modal multi-task MAE (MultiMAE) to reconstruct multiple input modalities simultaneously, and used a three-branch MAE to capture the spectral and spatial features of hyperspectral images, respectively (29). These studies indicate that in multi-view scenarios, MAE design must go beyond a single-modal reconstruction framework and systematically consider the collaborative modelling of cross-view information (30).

Finally, in Mask-AIR, we assign an independent MAE decoder to each view and perform reconstruction separately in feature space rather than uniformly in input space, a design that is consistent with the conventional MAE concept of “mask and reconstruct” extended to feature space. At the same time, the three decoders do not share weights, effectively preserving the unique attributes of each view, and fusion is achieved through an iterative attention mechanism, thereby avoiding the cross-view interference commonly encountered in multi-view MAE designs. This strategy of combining feature-space reconstruction with cross-attention fusion fully exploits the self-supervised learning advantages of MAE without introducing additional inference cost, laying a solid foundation for its excellent performance in the identification of small-sample functional foods such as *F. cirrhosa*.

### 4.2 Significance of data fusion for authenticity identification of FCB

In general, combining hyperspectral image data with other types of information can significantly improve the performance of most models, and the improvement tends to increase with the number of data sources fused. Existing studies have shown that multi-source fusion, by integrating optical, hyperspectral, radar and other data, provides richer feature sets and enhances discriminative ability. As a case in point, the hyperspectral imaging-based spectral-texture multi-view fusion strategy, combined with machine learning, enables efficient and non-destructive discrimination of *Pinellia ternate* and its counterfeits, achieving a classification accuracy of over 99% (31). However, data acquired from different sensors or through different processing methods contain both complementary information and redundant or interfering information. How to effectively preserve valuable information while eliminating unnecessary interference remains a key challenge in multi-modal learning. In this study, we adopt a cascaded design of 3D and 2D convolutions. Specifically, the 3D convolution reduces the dimensionality of hyperspectral bands while retaining critical spectral spatial features. A 2D convolution then extracts high-level semantic features that remain stable across views. Moreover, a downsampling convolution compresses the features of each view into a fixed number of tokens. This design not only provides a unified input format for subsequent MAE reconstruction but also allows us to systematically investigate the effect of token number on feature quality.

Experimental results show that the model achieves the best performance when fusing HSI, grayscale images, and PCA-generated pseudo-RGB images; in contrast, the two-view fusion that uses only grayscale images as the auxiliary view (Mask-AIR_HG) yields only limited improvement. This is because grayscale images provide only contour and intensity information, lacking sufficient colour and spectral discriminative power to form effective complementarity with other views. Therefore, fully exploiting complementary information from multi-view data, together with a sound feature extraction and tokenisation strategy, is a key route to enhancing model classification capacity and recognition accuracy.

### 4.3 Advantages of the Mask-AIR method over other models

Further comparison of performance differences among various models reveals that, although conventional CNN methods (e.g., 3D-CNN) outperform 2D-CNN, they still lag behind Transformer-based models such as ViT and SpectralFormer. Literature indicates that the performance of CNNs in handling complex three-dimensional data is limited, fundamentally due to the restricted receptive field of convolutional kernels and the difficulty of capturing context information (30, 32). Moreover, due to the inherent limitations of convolution operations, CNNs struggle to effectively exchange contextual information, while Transformers, although capable of solving this problem, suffer from high computational cost and poor performance on small datasets (33, 34). At the same time, models like ViT may introduce noise by over-attending to irrelevant features in real-world scenarios containing interfering information, which in turn degrades classification accuracy.

Based on these observations, the Mask-AIR method achieves systematic advantages through a set of well-designed components. In the feature extraction stage, the encoder uses non-shared parameters for each view, thereby preserving view-specific information. An MAE reconstruction module is also introduced, which randomly masks feature tokens with a high masking ratio and reconstructs the original features using mean squared error loss in the latent space. Existing studies have shown that the “mask-then-reconstruct” training strategy of the Transformer-based MAE enables the model to perform effectively on downstream tasks where labelled samples are few (35, 36). Furthermore, additional experiments have confirmed that even with an extremely small number of training samples, the HIS-MAE framework maintains a significant advantage over state-of-the-art methods (37). This self-supervised task forces the encoder to learn deep contextual relationships among tokens, which significantly enhances the robustness of feature representations. It is particularly suitable for the scarce-labelled data of FCB. Moreover, this module is active only during training and is completely removed during inference, so that no additional computational burden is incurred. To mitigate cross-modal interference caused by excessively high attention scores within a single view during multi-view fusion, we design an iterative cross-attention (Iter-Attn) mechanism. By alternately using different views as the query, key, and value, this method iteratively refines cross-view interactions and ensures a balanced contribution from each view. The synergy of the above designs enables Mask-AIR to outperform SpectralFormer by 4.25% in OA, 5.10% in AA, and 7.83% in Kappa coefficient. In summary, Mask-AIR effectively addresses the key challenges in authenticating FCB, scarcity of labelled data, cross-modal interference, and spectral feature variability, and provides a reliable and non-destructive technical solution for the identification of valuable functional foods.

### 4.4 Challenges and future prospects for FCB identification using Mask AIR

Despite the promising experimental results achieved by the proposed multi-view fusion models for rapid, non-destructive, and accurate FCB species identification, several limitations remain that warrant further investigation. A primary challenge lies in the data acquisition and annotation process. The framework requires accurately labelled hyperspectral data, which is both costly and time-consuming to collect. Furthermore, manual labelling is inherently subjective and prone to inconsistency, which can introduce noise into the training process and ultimately limit the overall classification performance (38). Similar issues have been observed in other spectroscopic and imaging applications, where the quality of ground-truth labels significantly influences model generalisability (39). To mitigate these labelling challenges, future work could explore unsupervised learning, self-supervised pre-training, or active learning strategies to reduce reliance on manually annotated samples (40, 41). Among these, YOLO-based automatic annotation is particularly promising, as deep detectors pre-trained on large-scale visual data can be adapted to generate initial labels for spectral-spatial samples, thereby substantially reducing human effort and accelerating dataset curation (42, 43).

Another significant concern is the high computational overhead of the proposed models, which may hinder their deployment in practical application environments with limited hardware resources. Complex architectures involving multi-view fusion and attention mechanisms often require substantial GPU memory and inference time, making real-time or edge-based applications difficult to realize. This is a recurring challenge in deep learning based spectral analysis, where the balance between model complexity and predictive accuracy requires careful consideration. To mitigate this issue, future research could investigate model compression techniques such as knowledge distillation, which transfers the knowledge from a large, complex teacher model to a more compact student network (44). For instance, EfficientSAM exemplifies this approach by distilling the heavy Segment Anything Model into a lightweight student encoder, achieving a 20-fold reduction in both parameters and inference time with only a minor drop in accuracy (23). Additionally, optimizing the attention mechanism itself, which can be achieved by employing sparse or linear attention variants, could significantly reduce computational cost without compromising accuracy. Similarly, EoMT demonstrates that by eliminating task-specific decoders and using a well-pretrained ViT with learnable queries, it can achieve up to a 75% reduction in inference latency while maintaining competitive performance, effectively reducing the overhead of attention-based interactions (24). The adoption of lightweight neural network architectures, such as MobileNet (45) or ShuffleNet (46), originally designed for mobile vision tasks, also offers a viable pathway toward efficient on-device inference.

## 5 Conclusion

In summary, the proposed Mask-AIR method, through masked autoencoder-guided feature-space information reconstruction and iterative attention-driven balanced multi-view fusion, successfully overcomes three major challenges in the authentication of *F. cirrhosa*: limited labelled data, cross-modal interference, and spectral feature variability. The method achieves state-of-the-art results on a self-built dataset comprising HSI, grayscale images, and PCA-generated pseudo-RGB images. Moreover, the framework is highly transferable and can be extended to the authenticity identification and quality grading of other valuable herbs (e.g., saffron, notoginseng, angelica), offering a non-destructive, efficient, and accurate tool for the quality control of natural products and functional foods.

Future work will be directed along several complementary avenues. One promising direction is the exploration of cross-modal masked prediction (e.g., predicting pseudo-RGB tokens from HSI tokens) to further strengthen semantic alignment and complementarity among multi-view data. Another important line of inquiry involves the integration of active learning and semi-supervised learning strategies to reduce dependence on expensive chemical labelling (e.g., HIS or NMR) and lower data annotation costs. Furthermore, inspired by recent advances in combining self-distillation with MAE models—where knowledge from a teacher network is distilled into the shallow layers of a student encoder, thereby enhancing the encoder’s ability to capture local details and edge features—we plan to incorporate self-distillation into the Mask-AIR framework. Existing studies have shown that integrating self-distillation into MAE pre-training can significantly enrich the representations of shallow encoder layers and notably improve downstream dense prediction tasks such as segmentation and object detection. This provides a promising direction for Mask-AIR to tackle more fine-grained analysis tasks on sliced *F. cirrhosa* samples, such as foreign object detection and region-of-interest segmentation. Lastly, the development of lightweight handheld hyperspectral devices, coupled with edge AI deployment, will enable rapid on-site non-destructive screening of medicinal and edible botanicals, contributing to intelligent quality assurance in the food and nutraceutical industries.

## Data Availability

The raw data supporting the conclusions of this article will be made available by the authors, without undue reservation.
